# Two new species of the ant genus *Acanthomyrmex* Emery, 1893 (Hymenoptera, Formicidae, Myrmicinae) from Thailand

**DOI:** 10.3897/zookeys.842.33609

**Published:** 2019-05-07

**Authors:** Weeyawat Jaitrong, Lamthai Asanok

**Affiliations:** 1 Thailand Natural History Museum, National Science Museum, Technopolis, Khlong 5, Khlong Luang, Pathum Thani, 12120, Thailand Thailand Natural History Museum, National Science Museum Pathum Thani Thailand; 2 Department of Agroforestry, Maejo University Phrae Campus, Phrae Province, 54140, Thailand Department of Agroforestry, Maejo University Phrae Campus Phrae Thailand

**Keywords:** *
Acanthomyrmex malikuli
*, *
Acanthomyrmex mizunoi
*, ant, distribution, taxonomy

## Abstract

*Acanthomyrmex* Emery, 1893 is a small myrmicine genus of the tribe Crematogastrini. Seventeen species of the genus have been recorded from Southeast Asia. Herein, two new species from Thailand (*Acanthomyrmexmalikuli***sp. n.** and *Acanthomyrmexmizunoi***sp. n.**) are added to this genus. Both species belong to *Acanthomyrmexluciolae* species group. *Acanthomyrmexmalikuli* was collected from hard dead wood on forest floor, while *A.mizunoi* nested in soil.

## Introduction

*Acanthomyrmex* Emery, 1893 is a small myrmicine genus of the tribe Crematogastrini (Ward et al. 2015; Blaimer et al. 2018). The genus was first described from Sri Lanka by Emery (1893) with *Acanthomyrmexluciolae* as the type species. Several ant taxonomists have published papers dealing with *Acanthomyrmex* after Emery (1893), including Wheeler (1919) (Malaysia), Wheeler (1930) (Taiwan), Moffett (1985) (Indonesia), Moffett (1986) (the Oriental region), Terayama (1995) (Thailand), Zhou and Zheng (1997) (China), Terayama et al. (1998) (Indonesia), and Eguchi et al. (2008) (Vietnam). Currently, 17 valid species are known in the Oriental region from India, Sri Lanka, the southernmost part of China, Taiwan to various countries in Southeast Asia (Bolton 1995; Antweb 2018; Table 1). None of them have not been found outside of this realm. In Thailand, only two species have been reported: *Acanthomyrmexferox* Emery, 1893 from southern part of Thailand (Jaitrong and Nabhitabhata 2005) and *Acanthomyrmexthailandensis* Terayama, 1995 from the northernmost part of Thailand (Terayama 1995).

We have examined the specimens of this genus from Thailand and recognized four species, two of which proved to be new to science. In this paper, we describe these two new species based on major and minor workers, dealate queen, and male. Keys to the Thai species based on the major and minor workers are provided.

**Table 1.** List of the *Acanthomyrmex* species and their distributions. Type localities are marked with *.

**Species**	**Distribution**
1. *Acanthomyrmexbasispinosus* Moffett, 1986	Sulawesi*
2. *Acanthomyrmexcareoscrobis* Moffett, 1986	Borneo (Sarawak*)
3. *Acanthomyrmexconcavus* Moffett, 1986	Borneo (Sarawak* and Sabah)
4. *Acanthomyrmexcrassispinus* Wheeler, 1930	Taiwan*
5. *Acanthomyrmexdusun* Wheeler, 1919	Borneo (Sarawak*)
6. *Acanthomyrmexferox* Emery, 1893	Thailand, Peninsular Malaysia (Perak*, Selangor and Pahang), Borneo (Sarawak, Sabah and Kalimantan) and Sumatra
7. *Acanthomyrmexfoveolatus* Moffett, 1986	Borneo (Sarawak*)
8. *Acanthomyrmexglabfemoralis* Zhou & Zheng, 1997	China (Guangxi*) and Vietnam
9. *Acanthomyrmexmalikuli* sp. n.	Thailand (Tak* and Uthai Thani Province)
10. *Acanthomyrmexmizunoi* sp. n.	Thailand (Chiang Rai, Nakhon Nayok* and Nakhon Ratchasima Provinces)
11. *Acanthomyrmexhumilis* Eguchi, Bui & Yamane, 2008	Vietnam*
12. *Acanthomyrmexlaevis* Moffett, 1986	Peninsular Malaysia (Perak*)
13. *Acanthomyrmexluciolae* Emery, 1893	Sri Lanka*
14. *Acanthomyrmexmindanao* Moffett, 1986	Philippines (Mindanao*)
15. *Acanthomyrmexminus* Terayama, Ito & Gobin, 1998	Sumatra*
16. *Acanthomyrmexnotabilis* (F. Smith, 1860)	Sulawesi, Seram, Batjan Island*
17. *Acanthomyrmexpadanensis* Terayama, Ito & Gobin, 1998	Sumatra*
18. *Acanthomyrmexsulawesiensis* Terayama, Ito & Gobin, 1998	Sulawesi*
19. *Acanthomyrmexthailandensis* Terayama, 1995	Thailand (Chiang Mai Province*)

## Materials and methods

The holotypes and paratypes of *Acanthomyrmexmalikuli* sp. n. and *Acanthomyrmexmizunoi* sp. n. are pin-mounted dry specimens. The holotypes and paratypes were examined for three *Acanthomyrmex* species in the *Acanthomyrmexluciolae* species group (*A.padanensis* Terayama, Ito & Gobin, 1998, *A.sulawesiensis* Terayama, Ito & Gobin, 1998, and *A.thailandensis* Terayama, 1995). A major and minor workers of *A.crassispinus* W.M. Wheeler, 1930 collected from the type locality in Taiwan were also examined. Most morphological observations were made with a ZEISS Discovery.V12 stereoscope.

Multi-focused montage images were produced using NIS-Elements-D-[Sequence6*-Focused] from a series of source images taken by a Nikon Digital Sight-Ri1 camera attached to a Nikon AZ100M stereoscope. Type specimens of each species were measured using an ocular micrometer recorded to the nearest 0.01 mm.

For a diagnosis of the genus, see Moffett (1986). The abbreviations used for the measurements and indices are as follows (edited from Eguchi et al. 2008):

**HL** Head length. Length of head proper, excluding mandibles, measured as a straight line from anterior clypeal margin to the mid-point of a line drawn across posterior margin of head.

**HW** Head width. Maximum width of head, in full-face view measured behind eyes (excluding eyes).

**EL** Eye length. Maximum length of eye with head positioned in profile view such that anterior and posterior eye margins are in same plane of focus.

**SL** Scape length. Maximum straight-line length of antennal scape excluding basal constriction and condylar bulb.

**MNH** Mesonotal height. Height of mesonotum of dealate queen and male, measured from highest point of mesonotum to lowest point of mesopleuron in lateral view.

**MSW** Mesoscutum width. Maximum width of mesoscutum of dealate queen and male.

**HFL** Hind femur length. Maximum length of hind femur, measured from junction with trochanter to junction with tibia.

**CI** Cephalic index. HW/HL × 100.

**SI** Scape index. SL/HW × 100.

**MNI** Mesonotal index. MSW/MNH × 100.

**MSI** Mesoscutal index. MSW/HW × 100.

**HFI** Hind femur index. HFL/HW × 100.

Abbreviations of the type depositories are as follows:

**AMK** Ant Museum, Faculty of Forestry, Kasetsart University, Thailand.

**MHNG**Muséum d’Histoire Naturelle, Genéva, Switzerland.

**SKYC** Seiki Yamane Collection, Kagoshima, Japan.

**THNHM** Natural History Museum of the National Science Museum, Thailand.

**USNM**The United States National Museum of Natural History, Smithsonian Institution, Washington, DC, USA.

## Results

### *Acanthomyrmexluciolae* species group

**Diagnosis of worker.** According to Moffett (1986), the *Acanthomyrmexluciolae* species group can be characterized by the following combination of characteristics of the worker: 1) posterior margin of head in major worker emarginate, so that the back of the head is distinctly bilobed in full-face view; 2) propodeal spiracle opening in major worker large and conspicuous; 3) basal funicular segments in both castes tending to be relatively slender (width of the second and third segments less than 25% greater than their average length in minor workers, and less than 50% in major workers); 4) hypostomal teeth invariably present in major worker; and 5) except in *A.crassispina*, first gastral tergite with numerous scattered hairs.

**Currently valid names.***Acanthomyrmexbasispinosus* Moffett, 1986; *A.crassispinus* W.M. Wheeler, 1930; *A.dusun* W.M. Wheeler, 1919; *A.ferox* Emery, 1893; *A.glabfemoralis* Zhou & Zheng, 1997; *A.laevis* Moffett, 1986; *A.luciolae* Emery, 1893; *A.padanensis* Terayama, Ito & Gobin, 1998; *A.sulawesiensis* Terayama, Ito & Gobin, 1998; *A.thailandensis* Terayama, 1995.

## Descriptions of new species

### Acanthomyrmex malikuli

Taxon classificationAnimaliaHymenopteraFormicidae

Jaitrong & Asanok sp. n.

http://zoobank.org/64713C37-7C8F-4A72-BAB8-CCE6888BDD1E

Figures 1 , 2 , 3

#### Type.

***Holotype major worker*** (THNHM-I-00124, THNHM), W Thailand, Tak Prov., Umphang Dist., Thung Yai Naresuarn East W.S., Thung Nanoi Forest Ranger Station, 15.50444°N, 98.95333°E, 18.II.2015, W. Jaitrong leg., colony no. TH15-WJT-321. ***Paratypes***: 1 dealate queen (THNHM-I-00115, THNHM), 3 major workers (THNHM-I-00121 to THNHM-I-00123, THNHM), and 56 minor workers (THNHM-I-00119 to THNHM-I-00120 and THNHM-I-00125 to THNHM-I-00178), same data as holotype (MHNG, SKYC, THNHM, USNM); 3 ergatoid queens (THNHM-I-01195, THNHM), 10 minor workers (THNHM-I-01196, THNHM, SKYC), 1 male (THNHM-I-01194, THNHM), W Thailand, Tak Prov., Umphang Dist., Thung Yai W.S., Bae Ki Station, 24.IX.2016, W. Jaitrong leg., colony no WJT240916-6.

#### Measurements.

***Holotype***: HL 2.41, HW 2.18, EL 0.17, SL 1.06, HFL 1.29, CI 111, EI 8, HFI 59, SI 48.

***Major workers*** (3 paratypes): HL 2.38–2.57, HW 2.08–2.24, EL 0.17–0.20, SL 1.09–1.12, HFL 1.29–1.32, CI 114–116, EI 8–9, HFI 59–62, SI 49–54. Non-type major workers (*n* = 5): HL 2.28–2.41, HW 2.01–2.15, EL 0.17–0.20, SL 0.99–1.09, HFL 1.16–1.25, CI 112–116, EI 8–10, HFI 57–60, SI 49–51.

***Minor workers*** (10 paratypes): HL 0.92–1.06, HW 1.06–1.19, EL 0.13–0.17, SL 0.92–0.99, HFL 1.02–1.06, CI 88–91, EI 12–15, HFI 89–100, SI 81–88.

***Dealate queen*** (paratype): HL 1.75, HW 1.95, EL 0.30, SL 1.06, HFL 0.96, MNH 168, MSW 1.39, CI 90, EI 15, HFI 68, MNI 82, MSI 71, SI 49. Non-type dealate queens (*n* = 4): HL 1.65–1.68, HW 1.85, EL 0.26–0.30, SL 0.92–0.96, HFL 1.29–1.32, MNH 162–168, MSW 1.35–1.39, CI 89–91, EI 14–16, HFI 68–71, MNI 82–84, MSI 72–74, SI 49–52.

***Ergatoid queen*** (3 paratypes): HL 1.98–1.05, HW 2.01–2.05, EL 0.23–0.26, SL 0.92–0.96, HFL 1.25, CI 98–100, EI 11–13, HFI 61–62, SI 46–47. Non-types (*n* = 2): HL 2.08–2.15, HW 2.05–2.08, EL 0.26, SL 0.96, HFL 1.25, CI 102–103, EI 13, HFI 61–62, SI 46–47.

***Male (paratype)***: HL 0.66, HW 0.83, EL 0.30, SL 0.20, HFL 1.02, MNL 0.99, MSW 0.89, CI 80, EI 36, HFI 124, MNI 90, MSI 108, SI 24. Non-types (*n* = 5): HL 0.69–0.73, HW 0.86–0.92, EL 0.33–0.36, SL 0.17–0.20, HFL 1.02–1.12, MNL 0.96–1.06, MSW 0.89–0.92, CI 78–82, EI 36–41, HFI 115–126, MNI 88–93, MSI 100–104, SI 19–22.

#### Description.

**Figure 1.**
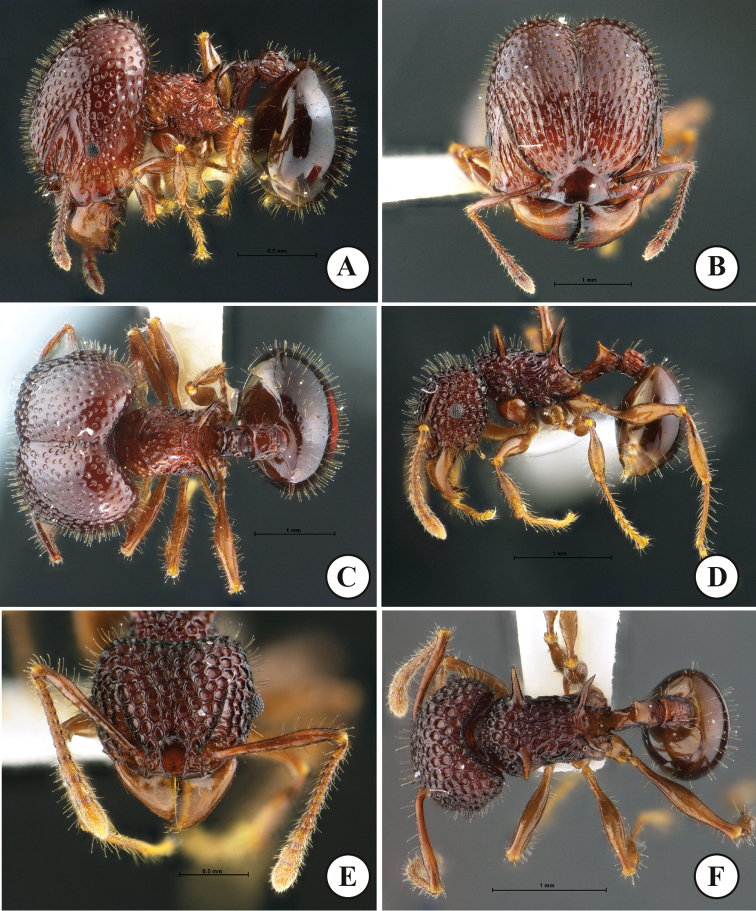
*Acanthomyrmexmalikuli* sp. n. **A–C** holotype major worker, THNHM-I-00124) **D–F** paratype minor worker, THNHM-I-01196 **A, D** body in profile **B, E** head in full-face view **C, F** dorsal view of body. Photos by Mr Yudthana Samung.

***Major worker*** (Fig. 1A–C). Head, in full-face view, longer than broad, lateral cephalic margins weakly convex and posterior cephalic margin shallowly concave medially; cephalic median furrow well-developed, extending anteriad to frontal area; in dorsal view, posterior half of head foveate with smooth and shiny interspaces, anterior half of head rugulose with dense foveae between wrinkles; foveae on posterior cephalic half smaller than those on anterior half; dorsum of head covered entirely with dense short erect hairs; mandible massive, subtriangular and subopaque; masticatory margin straight without distinct denticles; basal margin of mandible weakly concave; mandible with few hairs along masticatory margin; anterior margin of clypeus lacking medial and lateral setae, mid anterior margin of clypeus shallowly concave; median portion of clypeus smooth and shiny; frontal lobe poorly developed, only partly covering antennal socket; frontal carina conspicuous, reaching mid-length of head; antennal scrobe deep and conspicuous, with 3–5 irregular longitudinal ridges running from lateral portion of clypeus; eye relatively small, weakly convex, located at anterior ¼ of head length laterally; antenna 12-segmented with 3-segmented club; antennal scape thin (clavate, its apical portion narrower than antennal segments X–XII) and short, when laid backward not extending beyond mid-length of head; scape with more than 15 long erect hairs. Mesosoma short and stout; mesonotum almost straight, with sparse erect hairs; promesonotal suture present as weak groove dorsally; pronotal spine absent; metanotal groove indistinct; propodeal spine, in profile, relatively broad basally and almost straight apically, largely smooth and shiny, without erect hairs and decumbent hairs; legs entirely with numerous erect hairs; anterior face of each femur smooth and shiny, posterior face superficially reticulate with smooth and shiny interspaces. Petiole, in profile, with long anterior pedicel, usually a pair of posterolateral hairs present; petiolar node, in profile, subtriangular with sharp angle dorsally; posterior face of petiolar node with 2 or 3 pairs of erect hairs; in posterior view dorsum of petiolar node with pair of blunt angles, dorsal outline between angles weakly concave; postpetiole cylindrical, slightly shorter than high, in dorsal view with parallel sides and in profile with straight dorsal outline; postpetiole entirely rugose; dorsum of postpetiole with sparse erect hairs. Gaster smaller than head; gastral tergite I circular, smooth and shiny and with dense erect hairs. Body sculpture basically as in Figure 1A–C. Head, mesosoma, petiole and pospetiole reddish brown to dark brown; gaster darker than head and mesosoma, often black; mandible and legs reddish brown.

***Minor worker*** (Fig. 1D–F). Head, in full-face view, slightly shorter than broad, with convex lateral margins and weakly concave posterior margin; head entirely coarsely punctorecticulate, punctures large, 0.07–0.13 mm in diameter; cephalic dorsum entirely with dense erect hairs; mandible massive, subtriangular and superficially reticulate with smooth and shiny interspaces; masticatory margin with few ill-defined denticles; basal margin almost straight; mandible with few hairs along masticatory margin; anterior clypeal margin armed with 5 teeth, with long conspicuous median and lateral setae; median portion of clypeus with two distinct longitudinal ridges; frontal lobe poorly developed, partly concealing antennal socket; frontal carina conspicuous, reaching 2/3 of head length; antennal scrobe deep and conspicuous; eye relatively small, located anterior to mid-length of head, moderately protruding breaking outer margin of head; antenna 12-segmented with 3-segmented club; antennal scape slender, when laid backward surpassing cephalic corner by 1.5 times width of antennal scape; antenna with dense long erect hairs. Mesosoma, in profile, relatively stout, its dorsum sparsely with erect hairs; promesonotum convex and sloping gradually to metanotal groove; pronotum with a pair of long and sharp spines; promesonotal suture absent dorsally; metanotal groove present as inconspicuous broad impression just anterior to base of propodeal spine; propodeal spine in profile relatively long, its basal half straight and apical half gradually down-curved; propodeal spine largely smooth and shiny, without erect hairs or decumbent hairs; legs with numerous erect hairs; anterior face of each femur smooth and shiny, posterior face superficially reticulate with smooth and shiny interspaces. Petiole, in profile, with long anterior pedicel; petiolar node, in profile, moderately to strongly raised, with relatively angulate apex, in posterior view with concavity between acute lateral sharp spines or angles; posterior face of petiolar node with a pair of erect hairs; postpetiole rectangular, shorter than high, in dorsal view with parallel sides and in profile with straight dorsal outline; postpetiole entirely rugose, its dorsum with dense erect hairs. First gastral tergite suboval, smooth and shiny and with dense erect hairs. Body sculpture basically as in Figure 1D–F. Body entirely reddish brown to dark brown, gaster darker than head and mesosoma; mandible antenna and legs reddish brown to yellowish brown.

**Figure 2.**
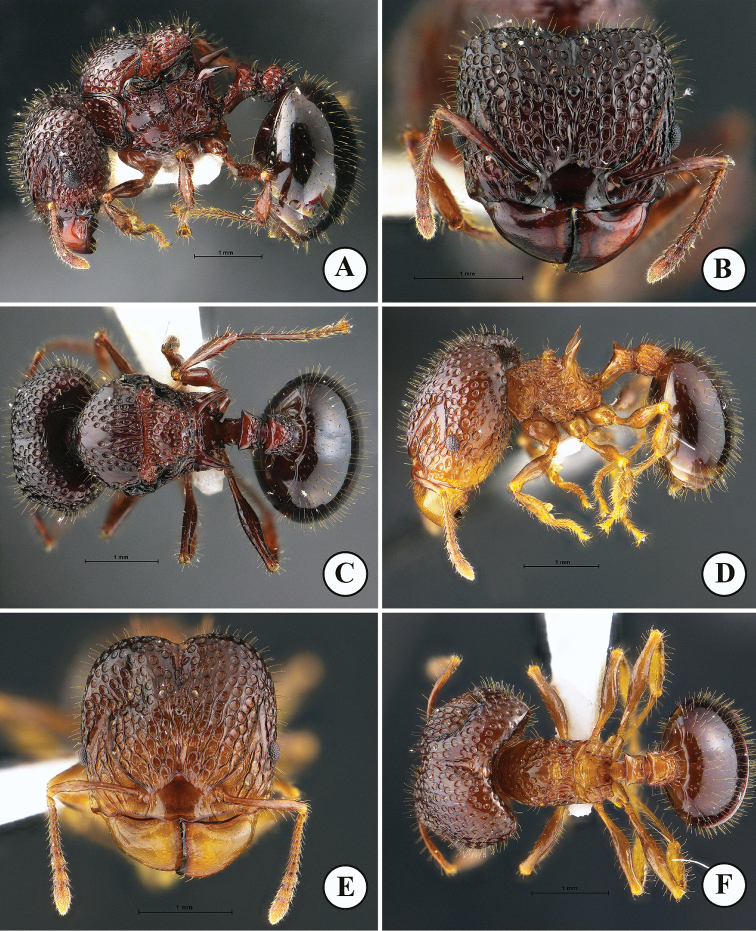
*Acanthomyrmexmalikuli* sp. n. **A–C** Paratype queen, THNHM-I-00115 **D–F** paratype ergatoid queen, THNHM-I-01195 **A, D** body in profile **B, E** head in full-face view **C, F** dorsal view of body. Photos by Mr Yudthana Samung.

***Dealate queen*** (Fig. 2A–C). Head, in full-face view, subrectangular, shorter than broad, with parallel sides and weakly concave posterior margin medially; cephalic median furrow well-developed, extending anteriad to frontal area; dorsum and lateral face of head foveate with smooth and shiny interspaces; dorsum of head entirely with dense erect hairs; mandible massive, subtriangular and superficially reticulate with smooth and shiny interspaces; masticatory margin straight with 2 or 3 small denticles near basal angle; basal margin weakly concave without denticles; median portion of anterior clypeal margin weakly produced anteriad, with weak concavity at middle of anterior portion, lacking medial and lateral setae; median portion of clypeus superficially reticulate with smooth and shiny interspaces; frontal lobe poorly developed, partly concealing antennal socket; frontal carina conspicuous, reaching 2/3 of head length; antennal scrobe deep and conspicuous; eye relatively large, convex, located anterior to mid-length of head; ocelli present, in full-face view median ocellus slightly larger than lateral ocelli and almost located at level of posterior margin of eye; antenna 12-segmented with 3-segmented club; antennal scape thin and short, when laid backward surpassing midlength of cephalic by 2 times width of antennal scape; scape with sparse long erect hairs. Mesosoma enlarged and high, its dorsum with erect hairs; pronotum in dorsal view narrow, without anterolateral spine; promesonotal suture distinct; scutellum in profile with convex dorsal outline, in dorsal view large and subtrapezoidal; mesoscutellum in dorsal view subrectangular with posterior margin weakly concave; mesoscutellum demarcated from mesoscutum by deep groove, in profile mesoscutellum protruding posteriorly; propodeal spine in profile relatively broad basally and weakly down-curved, smooth and shiny, without erect hairs and decumbent hairs; legs with sparse erect hairs, smooth and shiny. Petiole in profile with long anterior pedicel, petiolar node in profile with flat anterior face, weakly convex posterior face and acutely angulate dorsum; posterior face of petiolar node with 2 or 3 pairs of erect hairs; in posterior view dorsum of petiolar node with a pair of blunt angles, area between angles weakly convex; postpetiole rectangular, slightly shorter than high, in dorsal view with parallel sides and in profile with straight dorsal outline; postpetiole entirely rugose, its dorsum with sparse erect hairs. First gastral tergite circular, smooth and shiny, with dense erect hairs. Body sculpture basically as in Figure 2A–C. Head, mesosoma, petiole and pospetiole reddish brown to dark brown; gaster darker than head and mesosoma or often black; mandible and legs reddish brown.

***Ergatoid queen*** (Fig. 2D–F). Apterous. Similar to the major worker in structure, sculpture, coloration and pilosity, with the following conditions that should be noted: head slightly shorter and narrower (HW 2.02–2.05 mm, CI 102–103 in ergatoid queen; HW 2.08–2.24 mm, 112–116 in major worker); ocelli present, median ocellus in full-face view almost located at level of posterior margin of eye and almost as large as lateral ocelli; mesoscutellum protruding posteriorly. Body sculpture basically as in Figure 2D–F.

**Figure 3.**
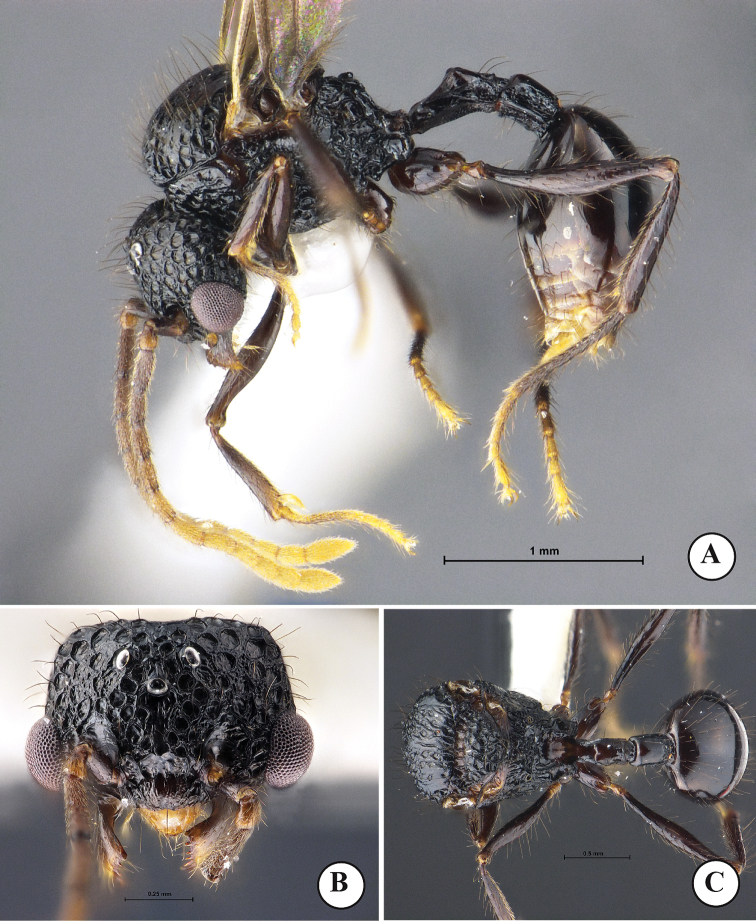
*Acanthomyrmexmalikuli* sp. n. (paratype male, THNHM-I-01194) **A** body in profile **B** head in full-face view **C** dorsal view of body. Photos by Mr Yudthana Samung.

***Male*** (paratype and non-types, Fig. 3A–C). Body black, but apical part of antenna, legs, and gaster reddish brown; wings light brown. Head in full-face view clearly broader than long, posterior margin almost straight; anterolateral part of head in which eye is located well produced laterally; mandible subtriangular, its masticatory margin with a large apical tooth, followed by 5 teeth including basal tooth; anterior clypeal margin roundly convex with weak median concavity; frontal lobe, frontal carina and antennal scrobe absent; ocelli present, with diameter less than distance between them; with head in full-face view median ocellus located a little behind level of posterior margin of eye; antenna 13-segmented, without club; antennal scape short when laid backward not reaching level of posterior margin of compound eye; antennal segment II very short; III much longer than broad and longer than each of segments IV–X. Mesosoma short and high; pronotal spine absent; mesoscutellum in profile strongly raised dorsoposteriad, in dorsal view with straight posterior margin; posterolateral part of propodeal dorsum in profile forming a blunt angle. Petiole in profile with long anterior pedicel; petiolar node in profile very low and bluntly raised; postpetiole clearly longer than high and cylindrical. Gaster relatively small and oval. Legs relatively long, smooth and shiny. Body sculpture basically as in Figure 3A–C.

#### Non-type material examined.

W Thailand, Tak Prov., Umphang Dist., Thung Yai Naresuarn East W.S., Thung Nanoi Forest Ranger Station, 18.II.2015, W. Jaitrong leg., colony no. TH15-WJT-342 (6 minor workers, 1 major worker, 3 ergatoid queens) (AMK, SKYC, THNHM); same locality, date and collector, TH15-WJT-337 (5 minor workers) (SKYC, THNHM); same locality, date and collector, colony no. TH15-WJT-334 (1 minor worker, 2 ergatoid queens) (THNHM); same locality and collector, 19.II.2018, colony no. WJT190218-5 (7 minor workers, 1 major worker, 1 ergatoid queen, 2 dealate queens) (MHNG, SKYC, THNHM), colony no. WJT190218-6 (5 minor workers, 2 ergatoid queens) (MHNG, SKYC, THNHM), colony no. WJT190218-7 (6 minor workers, 2 ergatoid queens) (SKYC, THNHM) and colony no. WJT190218-8 (6 minor workers, 2 major workers, 2 ergatoid queens) (THNHM); W Thailand, Tak Prov., Umphang Dist., Thung Yai Naresuarn East W.S., Bae Ki station – Thung Nanoi Station, 22.II.2018, W. Jaitrong leg., colony no. WJT220218-1 (7 minor workers, 1 major worker) (SKYC, THNHM), colony no. WJT220218-2 (14 minor workers, 1 major worker) (MHNG, SKYC, THNHM) and colony no. WJT220218-6 (22 minor workers, 1 major worker, 1 ergatoid queen) (MHNG, SKYC, THNHM); W Thailand, Tak Prov., Umphang Dist., Thung Yai Naresuarn East W.S., Huai Nam Kheao Station, 26.IX.2016, W. Jaitrong leg., colony no. WJT260916-1 (11 minor workers, 2 major workers, 1 dealate queen, 1 alate queen, 1 male) (USNM, SKYC, THNHM); W Thailand, Tak Prov., Umphang Dist., Thung Yai Naresuarn East W.S., Ka-ngae Sod Forest Ranger Station, 19.II.2015, W. Jaitrong leg., colony no. TH15-WJT-359 (7 minor workers, 2 major workers, 1 dealate queen) (SKYC, THNHM); same locality, date and collector, TH15-WJT-360 (3 minor workers, 1 major worker, 1 dealate queen) (SKYC, THNHM); same locality and collector, 21.IX.2014, W. Jaitrong leg., colony no. WJT210914-5A (5 minor workers) (THNHM); W Thailand, Tak Prov., Umphang Dist., Thung Yai Naresuarn East W.S., Bae Ki Station, 24.IX.2016, W. Jaitrong leg., colony no. WJT240916-8 (1 minor worker, 1 major workers, 1 alate queen, 1 male) (THNHM); W Thailand, Tak Prov., Umphang Dist., Thung Yai Naresuarn East W.S., Yuyi junction, 25.IX.2016, W. Jaitrong leg., colony no. WJT250916-3 (3 minor workers, 1 major worker) (THNHM); W Thailand, Tak Prov., Umphang Dist., Thung Yai Naresuarn East W.S., Utakae station, 23.IX.2016, W. Jaitrong leg., colony no. WJT230916-1 (9 minor workers, 2 major workers, 1 male) (THNHM); W Thailand, Tak Prov., Umphang Dist., Thung Yai Naresuarn East W.S., Ka Ngaeki, Head Quarter (HQ) 22.IX.2016, W. Jaitrong leg., colony no. WJT220916-1 (12 minor workers, 3 major workers, 1 male) (USNM, SKYC, THNHM); central Thailand, Uthai Thani Prov., Ban Rai Dist., 23.IX.2014, W. Jaitrong leg., colony no. WJT230914-15 (8 minor workers, 1 ergatoid queen) (THNHM); W Thailand, Tak Prov., Umphang Dist., Thung Yai Naresuarn East W.S., Ka Ngaeki, Head Quarter (HQ) 27.IX.2016, W. Jaitrong leg., colony no.WJT270916-4 (2 minor workers, 1 alate queen, 8 males) (THNHM); W Thailand, Tak Prov., Umphang Dist., Thung Yai Naresuarn East W.S., Huai Nam Kheao Station, 26.IX.2016, W. Jaitrong leg., colony no.WJT260916-1 (3 minor workers, 1 ergatoid, 1 male) (THNHM).

#### Etymology.

The specific name is dedicated to Mr Vichai Malikul, Scientific Illustrator, Department of Entomology, National Museum of Natural History, Smithsonian Institution, Washington, D.C. (USNM, U.S.A.), who kindly helped W. Jaitrong when he visited the USNM.

#### Bionomics.

*Acanthomyrmexmalikuli* sp. n. was found to nest in preformed cavities in hard dead wood on the forest floor in a lower tropical mountain forest (800–1000 m a.s.l.). Colony composition and behavior of this species will be reported in a different paper by Jaitrong and others.

#### Distribution.

Thailand (Tak and Uthai Thani Provinces).

#### Remarks.

*Acanthomyrmexmalikuli* can be distinguished from all other species in *A.luciolae* group by the following combination of characters: 1) major and minor workers having dark brown to black body; 2) dorsum of head (in profile view) strongly convex in both castes; 3) major worker having anterior half of head rugulose with dense foveae between wrinkles; and 4) major and minor workers with dense erect hairs on the head and first gastral tergite. This species is most closely related to *A.mizunoi* (see “Remarks” under *A.mizunoi*). The new species can be easily distinguished from *A.padanensis* by the following characteristics: 1) in major worker, mid anterior margin of clypeus shallowly concave (anterior clypeal margin weakly convex in *A.padanensis*); 2) in major worker, head with denes erect hairs (without hairs in *A.padanensis*); and 3) in ergatoid queen, without pronotal spine, general shape as in major worker (with pronotal spine, general shape as in minor worker). This species is separated from *A.sulawesiensis* by the presence of pronotal spine in ergatoid queen (without pronotal spine in *A.malikuli*; with pronotal spine in *A.sulawesiensis*); and major worker having anterior half of head rugulose with dense foveae between wrinkles (entire of head lacking rugae in *A.sulawesiensis*). *Acanthomyrmexmalikuli* differs from *A.dusun* Wheeler, 1919 as follows: 1) anterior half of head rugulose with dense foveae between wrinkles in *A.malikuli* (lacking rugae in *A.dusun*); and 2) propodeal spine almost straight in *A.malikuli* (short, cylindrical, curved ventrad in *A.dusun*).

### Acanthomyrmex mizunoi

Taxon classificationAnimaliaHymenopteraFormicidae

Jaitrong & Asanok sp. n.

http://zoobank.org/F5FCA88F-E507-420D-8FA1-84383C83ECDE

Figures 4 , 5 , 6

#### Type.

***Holotype major worker*** (THNHM-I-01197, THNHM), central Thailand, Nakhon Nayok Prov., Muang Dist., Ban Hin Tang, 14.40611°N, 101.37139°E, 22.X.2017, W. Jaitrong leg., colony no. WJT221017-12. ***Paratypes***: 5 major workers (THNHM-I-01198, MHNG, SKYC, THNHM), 64 minor workers (THNHM-I-01199, MHNG, SKYC, THNHM, USNM) and 3 dealate queens (THNHM-I-01200, SKYC, THNHM), same data as holotype; 4 ergatoid queens (THNHM-I-00054, THNHM-I-00061, THNHM-I-00063, THNHM-I-00065, SKYC, THNHM), 8 minor workers (THNHM-I-00055, THNHM-I-00057 to THNHM-I-00060, THNHM-I-00062, THNHM-I-00064, THNHM-I-00066, SKYC, THNHM, USNM), and 1 male (THNHM-I-00056), NE Thailand, Nakhon Ratchasima Prov., Pak Chong Dist., dry evergreen forest, 31.V.2000, W. Jaitrong leg., colony no. WJT310500-1; 1 ergatoid queen (THNHM-I-0053, THNHM) and 1 male (AMK007, THNHM), NE Thailand, Nakhon Ratchasima Prov., Khao Yai N.P., hill evergreen forest, 30.V.2000, C. Bourmas leg.

#### Measurements and indices.

***Holotype major***: HL 2.67, HW 2.24, EL 0.23, SL 1.06, HFL 1.29, CI 84, EI 10, HFI 57, SI 47.

***Major workers*** (6 paratypes): HL 2.48–2.67, HW 2.05–2.28, EL 0.17–0.20, SL 0.96–1.02, HFL 1.22–1.35, CI 83–85, EI 8–9, HFI 56–60, SI 45–47.

***Minor workers*** (10 paratypes): HL 0.96–1.02, HW 1.02–1.09, EL 0.13–0.17, SL 0.79–0.86, HFL 0.96–1.02, CI 106–110, EI 12–16, HFI 91–97, SI 73–81.

***Dealate queens*** (3 paratypes): HL 1.78–1.82, HW 1.98–2.01, EL 0.23, SL 0.92–0.96, HFL 1.35–1.39, MNL 1.75–1.78, MSL 1.39–1.42, CI 109–111, EI 11–12, HFI 68–70, MNI 79–80, MSI 70–72, SI 47–48.

***Ergatoid queen*** (4 paratypes): HL 2.18–2.24, HW 1.95–2.01, EL 0.20, SL 0.92–0.96, HFL 1.19–1.25, CI 89–91, EI 10, HFI 60–63, SI 47–48.

***Male (paratype)***: HL 0.69, HW 0.86, EL0.30, SL 0.17, HFL 1.06, MNL 0.96, MSW 0.83, CI 124, EI 35, HFI 123, MNI 86, MSI 96, SI 19. Non-type (*n* = 5): HL 0.66–0.73, HW 0.86–0.89, EL 0.26–0.30, SL 0.17–0.20, HFL 1.0–1.06, MNL 0.96–0.99, MSW 0.86–0.89, CI 123–130, EI 30–33, HFI 115–119, MNI 90, MSI 96–100, SI 19–22.

#### Description.

**Figure 4.**
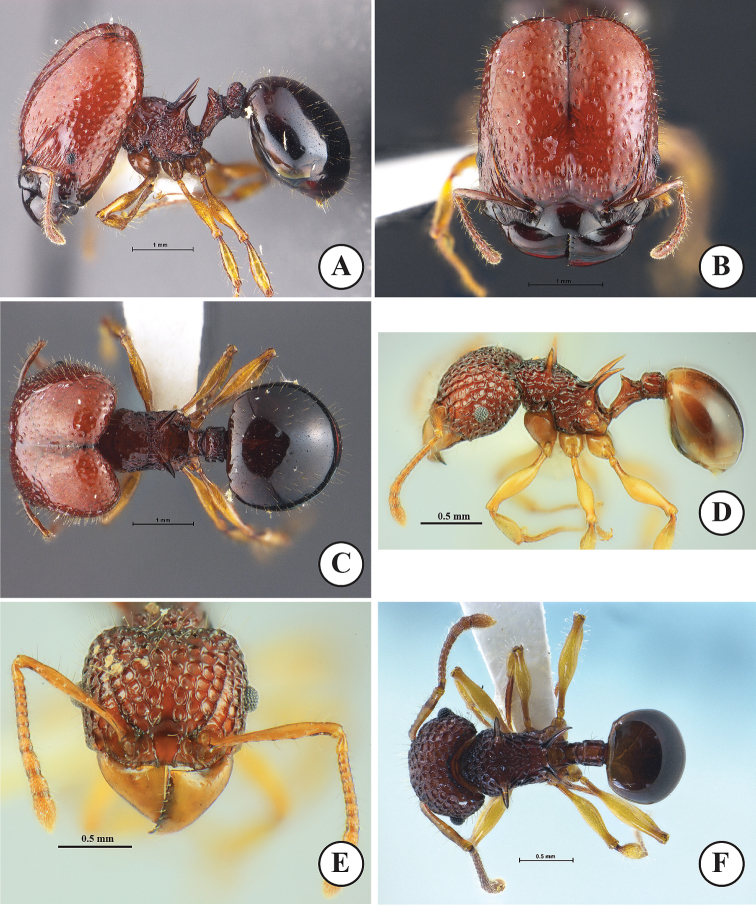
*Acanthomyrmexmizunoi* sp. n. Holotype major worker, THNHM-I-01197 **D–F** paratype minor worker, THNHM-I-01199 **A, D** body in profile **B, E** head in full-face view **C, F** dorsal view of body. Photos by Mr Yudthana Samung (**A–C**) or Weeyawat Jaitrong (**D–F**).

***Major worker*** (Fig. 4A–C). Head, in full-face view, clearly longer than broad, lateral cephalic margins straight, posterior cephalic corners rounded, and posterior cephalic margin shallowly concave medially; cephalic median furrow well developed, extending anteriad to frontal area; dorsum of head entirely with dense erect hairs; mandible massive, subtriangular and superficially reticulate with smooth and shiny interspaces; masticatory margin without distinct denticles; basal margin weakly concave; mandible with few hairs along its masticatory margin and outer margin; median portion of anterior clypeal margin weakly produced anteriad, produced portion with anterior margin weakly concave at middle; clypeus lacking medial and lateral setae; median portion of clypeus smooth and shiny; frontal lobe poorly developed, partly concealing antennal socket; frontal carina conspicuous, reaching midlength of head; antennal scrobe deep and conspicuous, with 8–10 longitudinal ridges running from lateral portion of clypeus; eye relatively small, weakly convex, located at anterior ¼ of head length laterally; antenna 12-segmented with 3-segmented club; antennal scape thin and short, not extending beyond midlength of head; scape with more than 15 long erect hairs. Mesosoma short and stout; pronotal spine absent; mesonotum weakly convex, with sparse erect hairs; promesonotal suture present as a weak groove dorsally; metanotal groove present as inconspicuous, broad impression just in front of base of propodeal spine; propodeal spine in profile relatively broad basally and sharp apically, largely smooth and shiny, without erect hairs and decumbent hairs; dorsum of each femur without erect hairs; legs smooth and shiny. Petiole in profile with long anterior pedicel, usually a pair of posterolateral hairs present; petiolar node, in profile, subtriangular with blunt angle dorsally; posterior face of petiolar node with 2 or 3 pairs of erect hairs; in posterior view dorsum of petiolar node with a pair of blunt angles, dorsal outline between angles strongly concave; postpetiole clearly shorter than high, in dorsal view rectangular with parallel sides and in profile with convex dorsal outline; dorsum of postpetiole with sparse erect hairs; postpetiole entirely rugose. Gaster smaller than head; gastral tergite I circular, smooth and shiny and with dense erect hairs. Body sculpture basically as in Figure 4A–C. Head, mesosoma, petiole, and postpetiole reddish brown; gaster darker than head, mesosoma and waist often black; mandible and legs reddish brown but paler than head.

***Minor worker*** (Fig. 4D–F). Head, in full-face view, slightly shorter than broad, with slightly convex sides and strongly concave posterior margin; head entirely coarsely punctorecticulate, punctures large, 0.07–0.13 mm in diameter; dorsum of head entirely with dense erect hairs; mandible massive, subtriangular and superficially reticulate with smooth and shiny interspaces; masticatory margin with few ill-defined denticles; basal margin almost straight; mandible with few hairs along its masticatory margin and outer margin; anterior clypeal margin armed with several teeth, with conspicuous median and lateral setae; median portion of clypeus with two distinct longitudinal ridges; frontal lobe poorly developed, partly concealing antennal socket; frontal carina conspicuous, reaching 2/3 of head length; antennal scrobe deep and conspicuous; compound eye relatively small, moderately protruding, located anterior to mid-length of head; antenna 12-segmented with 3-segmented club; antennal scape slender, when laid backward surpassing posterolateral corner of head by 1.2 times width of antennal scape; antenna with dense long erect hairs. Mesosoma in profile relatively stout, its dorsum sparsely with erect hairs; pronotum with a pair of straight spines, which are clearly shorter than propodeal spine; promesonotum convex and sloping gradually to metanotal groove; promesonotal suture absent dorsally; metanotal groove present as inconspicuous broad impression just anterior to base of propodeal spine; propodeal spine in profile relatively long and slender, gradually down-curved, largely smooth and shiny, without erect hairs and decumbent hairs; all femora smooth and shiny, dorsally without erect hairs. Petiole in profile with long anterior pedicel; petiolar node in profile moderately to strongly raised, with relatively angulate apex, in posterior view with strong concavity between acute lateral sharp spines or angles; posterior face of petiolar node with a pair of erect hairs; postpetiole rectangular, almost as long as high, in dorsal view with parallel sides and in profile with straight dorsal outline; postpetiole entirely rugose, its dorsum with sparse erect hairs. First gastral tergite suboval, smooth and shiny and with less than 10 erect hairs around anterior portion. Body sculpture basically as in Figure 4D–F. Body entirely yellowish brown to reddish brown, gaster darker than head and mesosoma; mandible, antenna and legs yellowish brown to reddish brown but paler than head.

**Figure 5.**
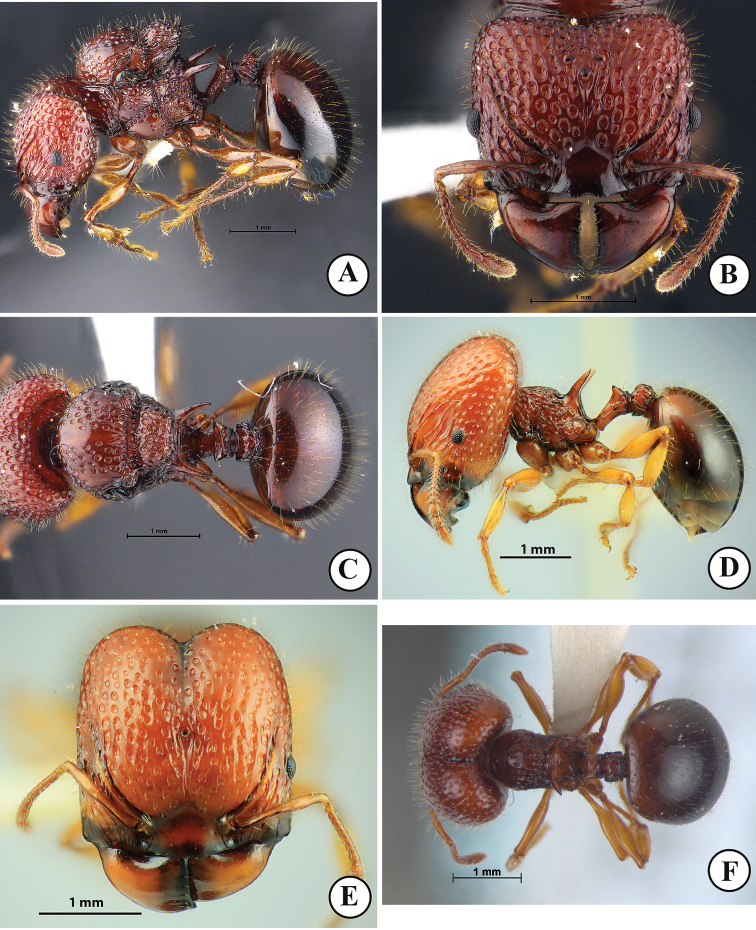
*Acanthomyrmexmizunoi* sp. n. **A–C** paratype dealate queen, THNHM-I-01200 **D–F** paratype ergatoid queen, THNHM-I-00053 **A, D** body in profile **B, E** head in full-face view **C, F** dorsal view of body. Photos by Mr Yudthana Samung (**A–C**) or Weeyawat Jaitrong (**D–F**).

***Dealate queen*** (Fig. 5A–C). Head, in full-face view, subrectangular, shorter than broad, slightly broader posteriorly, with concave posterior margin; cephalic median furrow well developed, extending anteriad to frontal area; dorsum and lateral face of head foveate with smooth and shiny interspaces; dorsum of head entirely with dense erect hairs; mandible massive, subtriangular and superficially reticulate with smooth and shiny interspaces; masticatory margin straight with 3 or 4 small denticles near basal angle; basal margin weakly concave without denticles; median portion of anterior clypeal margin weakly produced anteriad, anterior margin of the produced portion concave at middle, with medial seta; median portion of clypeus smooth and shiny; frontal lobe poorly developed, partly concealing antennal socket; frontal carina conspicuous, reaching 2/3 of head length; antennal scrobe deep and conspicuous; eye relatively large, convex, located anterior to midlength of head; ocelli present, median ocellus, in full-face view, almost as long as lateral ocelli and almost located at level of posterior margin of eye; antenna 12-segmented with 3-segmented club; antennal scape thin and short, when laid backward surpassing midlength of head by 2.5 times width of antennal scape; scape with sparse long erect hairs. Mesosoma enlarged and high, its dorsum with erect hairs; pronotum narrow, without spine; promesonotal suture distinct; scutellum in profile with strongly convex dorsal outline, in dorsal view large and subtrapezoidal; mesoscutellum in dorsal view subrectangular with posterior margin weakly concave, demarcated from mesoscutum by deep groove, mesoscutellum protruding posteriorly and overhanging narrowed metanotum; propodeal spine in profile relatively broad basally and weakly down-curved, smooth and shiny, without erect hairs and decumbent hairs; dorsum of each femur without erect hairs; legs smooth and shiny. Petiole in profile with long anterior pedicel, 1 or 2 pairs of posterolateral hairs present; petiolar node in profile subtriangular with blunt angle dorsally; posterior face of petiolar node with 2 or 3 pairs of erect hairs; in posterior view dorsum of petiolar node with a pair of blunt angles, dorsal outline between angles strongly concave; postpetiole clearly shorter than high, in dorsal view rectangular with parallel sides and in profile with convex dorsal outline; dorsum of postpetiole with sparse erect hairs; postpetiole entirely rugose. First gastral tergite oval, smooth and shiny, with dense erect hairs. Body sculpture basically as in Figure 5A–C. Head, mesosoma, petiole, and pospetiole reddish brown to dark brown; gaster darker than head and mesosoma, often black; mandible and legs reddish brown, always paler than head.

***Ergatoid queen*** (Fig. 5D–F). Apterous. Similar to the major worker in structure, sculpture, coloration and pilosity, with the following conditions that should be noted: relatively smaller (HW 1.95–2.01 mm in ergatoid queen, 2.05–2.28 mm in major worker); median ocellus present; lateral ocelli absent; body colour paler than in major worker. Body sculpture basically as in Figure 5D–F.

**Figure 6.**
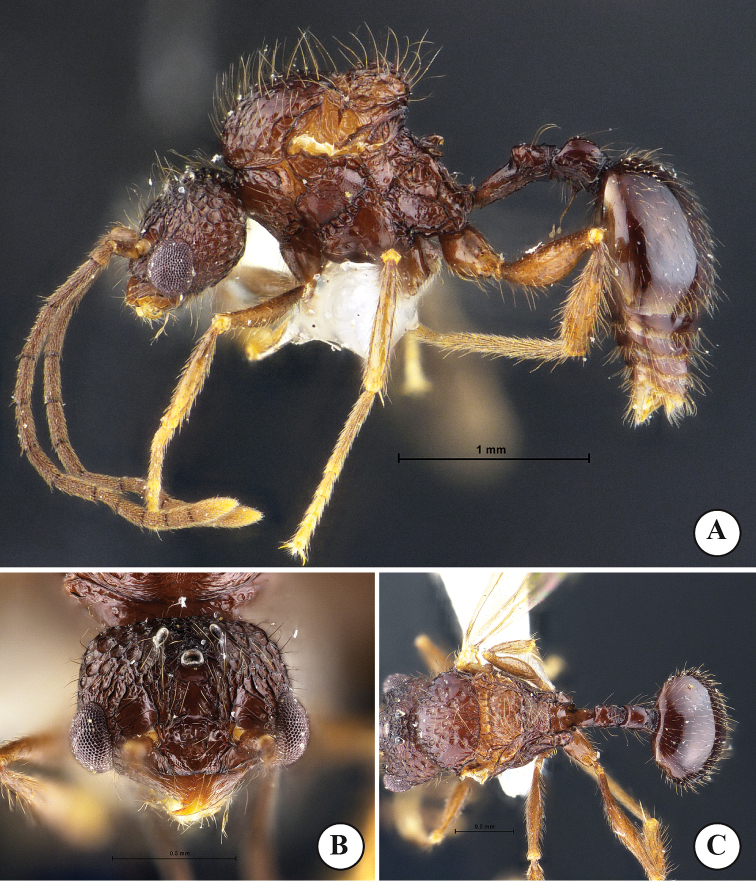
*Acanthomyrmexmizunoi* sp. n. (paratype male, THNHM-I-00056) **A** body in profile **B** head in full-face view **C** dorsal view of body. Photos by Mr Yudthana Samung.

***Male*** (Fig. 6A–C). Body dark brown, but apical part of antenna, legs, and gaster reddish brown; wings light brown. Head, in full-face view, broadened-subpentagonal, posterior margin almost straight; anterolateral part of head in which compound eye is located well produced laterally; mandible subtriangular, masticatory margin with large apical tooth, followed by 5 teeth including basal tooth; anterior clypeal margin roundly convex; frontal lobe, frontal carina and antennal scrobe absent; ocelli present, with diameter less than distance between them; median ocellus, in full-face view, located slightly behind level of posterior margin of eye and slightly larger than lateral ocelli; antenna 13-segmented, without club; antennal scape short, when laid backward not reaching level of posterior margin of eye; antennal segment II very short; III much longer than broad and longer than each of segments IV–X; IX narrower and shorter than VIII and X. Mesosoma short and high; pronotal spine absent; mesoscutellum in profile strongly raised posterodorsally, in dorsal view with straight posterior margin, demarcated from mesoscutum by deep and broad groove; mesoscutellum strongly convex; posterolateral part of propodeal dorsum in profile forming a blunt angle. Petiole, in profile, with long anterior pedicel; petiolar node in profile very low and bluntly raised; postpetiole almost as long as high and its dorsal outline convex. Gaster relatively small and oval. Legs relatively long, smooth and shiny. Body sculpture basically as in Figure 6A–C.

#### Non-type material examined.

N Thailand, Chiang Rai Prov., Huai Pong Coffee Plantation, 19.08917°N, 99.36250°E, 31.VIII.2016, R. Mizuno leg., colony no. RM-65 (10 minor workers, 4 ergatoid queens, 4 males) (THNHM); NE Thailand, Nakhon Ratchasima Prov., Pak Chong Dist., Hill Evergreen Forest, 30.V.2000, C. Boumas leg., general collection (1 male) (THNHM); central Thailand, Nakhon Nayok Prov., Muang Dist., Ban Hin Tang, 14.40611°N, 101.37139°E, 22.X.2017, W. Jaitrong leg., colony no. WJT221017-8 (1 major worker and 1 major worker) (THNHM); central Thailand, Nakhon Nayok Prov., Muang Nakhon Nayok, Ban Hin Tang [Ban in Tang], 70 m a.s.l., 7.VII.2018, W. Jaitrong leg., colony no. WJT070718-10 (1 male); central Thailand, Nakhon Nayok Prov., Muang Dist., Hill evergreen forest, 8.VII.2018, W. Jaitrong leg., colony no. WJT080718-3 (6 minor workers, 4 major workers, 1 dealate queen, 1 male) (SKYC, THNHM).

#### Bionomics.

*Acanthomyrmexmizunoi* inhabits highlands and nests in soil. The type series and non-types were collected from Nakhon Nayok and Nakhon Ratchasima Provinces in a primary hill evergreen forest (ca 800 m a.s.l.). A colony (Colony no. RM-65) from the Chiang Rai Province, northern Thailand was collected in a coffee plantation (ca 900 m a.s.l.).

#### Etymology.

The specific name is dedicated to Mr Riou Mizuno (Kagawa University, Japan), who confirmed the ergatoid queen of this new species and donated to us the specimens collected from the Chiang Rai Province.

#### Distribution.

Thailand (Chiang Rai, Nakhon Nayok and Nakhon Ratchasima Provinces).

#### Remarks.

**Figure 7.**
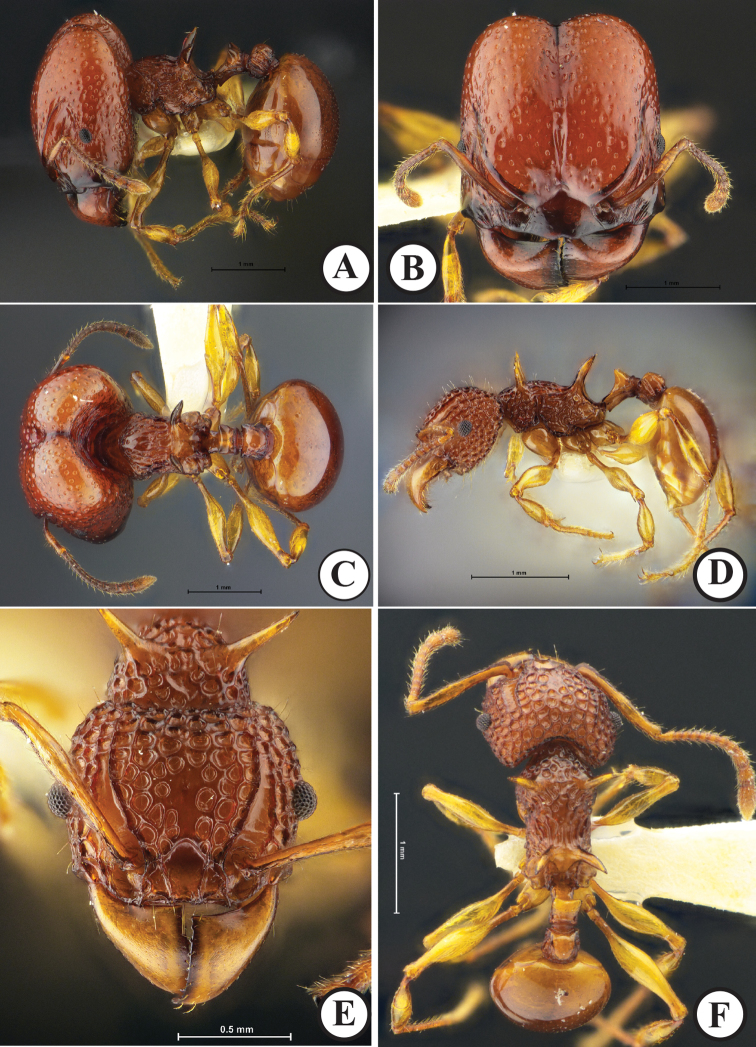
*Acanthomyrmexcrassispinus***A–C** Non-type major worker (THNHM-I-05475) **D–F** Non-type minor worker (THNHM-I-05476) **A, D** body in profile **B, E** head in full-face view **C, F** dorsal view of body. Photos by Mr Yudthana Samung.

*Acanthomyrmexmizunoi* can be distinguished from all other species in *A.luciolae* group by the following combination of characters: 1) major and minor workers having reddish brown to dark brown body; 2) in major worker, anterior dorsum of head lacking rugae; 3) major worker with sparse shallow foveae on dorsum of head; 4) head with dense erect hairs in both castes; 5) major worker without erect hairs on dorsum of each femur; and 6) minor worker with fewer than 10 erect hairs around anterior portion of first gastral tergite. This species is most similar to *A.malikuli*. However, it is easily distinguishable from the latter by the following characteristics: 1) in the major worker, anterior dorsum of head lacking rugae in *A.mizunoi* (rugulose in *A.malikuli*); 2) postpetiole clearly shorter than high in *A.mizunoi* (almost as long as high, with straight dorsal outline in *A.malikuli*); 3) first gastral tergite with very short appressed hairs in *A.mizunoi* (without very short appressed hairs in *A.malikuli*); 4) dorsal and lateral faces of femora without hairs in *A.mizunoi* (with erect hairs in *A.malikuli*); 5) in the minor worker, first gastral tergite without or with 1–4 erect hairs in *A.mizunoi* (with dense erect hairs in *A.malikuli*); 6) in the ergatoid queen, mesoscutellum not protruding posteriorly in *A.mizunoi* (protruding posteriorly and overhanging narrow metanotum in *A.malikuli*); 7) in the male, mesopleuron almost smooth in *A.mizunoi* (strongly sculptured in *A.malikuli*); and 8) postpetiole in profile clearly shorter than high in *A.mizunoi* (as long as high and cylindrical in *A.malikuli*). *Acanthomyrmexmizunoi* is also similar to *A.crassipinus* Wheeler, 1930 in terms of the general appearance of the minor worker. After careful examination of the holotype and paratype workers of *A.mizunoi* with non-type workers of *A.crassispinus* from Taiwan, Orchid Island (Fig. 7), 13.III.2017, collected by P.C. Hsu, we concluded that *A.mizunoi* can be easily distinguished from the latter by the following characteristics: 1) major worker with dense erect hairs on the head and first gastral tergite in *A.mizunoi* (completely without hairs in *A.crassipinus*); 2) propodeal spine thin and cylindrical in *A.mizunoi* (flat and broad at the base in *A.crassipinus*); 3) minor worker having a petiole and postpetiole with sparse erect hairs in *A.mizunoi* (without hairs in *A.crassipinus*); and 4) first gastral tergite with 1–4 erect hairs in *A.mizunoi* (without hairs in *A.crassipinus*). A specimen (CASENT0101994ANTWEB; images available on Antweb), collected from Khao Yai National Park, Thailand, has been identified as *A.crassispinus*. Images of this specimen agree well with the minor worker of *A.mizunoi* in having dense erect hairs on the head, with sparse erect hairs on the petiole and postpetiole. Thus, this specimen (CASENT0101994ANTWEB) should be reidentified as a non-type of our new species, *A.mizunoi*. The new species can be easily distinguished from *A.padanensis* by the following characteristics: 1) in major worker, mid anterior margin of clypeus shallowly concave (anterior clypeal margin weakly convex in *A.padanensis*); 2) in major worker, head with denes erect hairs (without hairs in *A.padanensis*); and 3) in ergatoid queen, without pronotal spine, general shape as in major worker (with pronotal spine, general shape as in minor worker in *A.padanensis*).

##### Key to Thai species based on major worker

**Table:** 

1	Dorsum of head with conspicuous alveolate sculpture	**2**
–	Dorsum of head virtually free of sculpture, smooth and shiny	*** A. thailandensis ***
2	Petiole with long, cylindrical spines laterally on apex of node; smaller species (HW 1.68–1.82 mm)	*** A. ferox ***
–	Such spines absent; larger species (HW > 2.05 mm)	**3**
3	Anterior half of dorsum of head rugulose; postpetiole almost as long as high, with straight dorsal outline; first gastral tergite without very short appressed hairs; dorsal and lateral faces of femora with erect hairs	***A.malikuli* sp. n.**
–	Anterior half of dorsum of head without rugae; postpetiole clearly shorter than high, with convex dorsal outline; first gastral tergite densely with very short appressed hairs; dorsal and lateral faces of femora without erect hairs	***A.mizunoi* sp. n.**

##### Key to Thai species based on minor worker

**Table:** 

1	Petiole with long, cylindrical spines laterally on apex of node; postpetiole almost smooth	*** A. ferox ***
–	Such spines absent; postpetiole strongly sculptured	**2**
2	First gastral tergite with 0–4 erect hairs	***A.mizunoi* sp. n.**
–	First gastral tergite with more than 10 erect hairs	**3**
3	First gastral tergite with about 10 relatively short erect hairs occurring only in anterior half of the segment; lateral and dorsal faces of femora lacking hairs; body reddish brown (brighter)	*** A. thailandensis ***
–	First gastral tergite densely with relatively long erect hairs occurring over surface; lateral and dorsal faces of mid and hind femora with sparse hairs; body dark brown (darker)	***A.malikuli* sp. n.**

## Supplementary Material

XML Treatment for Acanthomyrmex malikuli

XML Treatment for Acanthomyrmex mizunoi
